# Characterization of a novel sRNA contributing to biofilm formation in *Salmonella enterica* serovar Typhimurium

**DOI:** 10.17912/micropub.biology.000796

**Published:** 2023-04-20

**Authors:** Sayema Naaz, Najmuj Sakib, Dominika Houserova, Rani Badve, Aline Crucello, Glen M Borchert

**Affiliations:** 1 Department of Pharmacology, College of Medicine, University of South Alabama, Mobile, AL

## Abstract

Small RNAs (sRNAs) are short noncoding RNAs of ~50-200 nucleotides believed to primarily function in regulating crucial activities in bacteria during periods of cellular stress. This study examined the relevance of specific sRNAs on biofilm formation in nutrient starved *Salmonella enterica* serovar Typhimurium. Eight unique sRNAs were selected for deletion primarily based on their genomic location and/or putative targets. Quantitative and qualitative analyses confirm one of these, sRNA1186573, is required for efficient biofilm formation in *S. enterica* further highlighting the significance of sRNAs during *Salmonella* stress response.

**Figure 1. Characterization of a novel sRNA that regulates biofilm formation in Salmonella enterica serovar Typhimurium.**
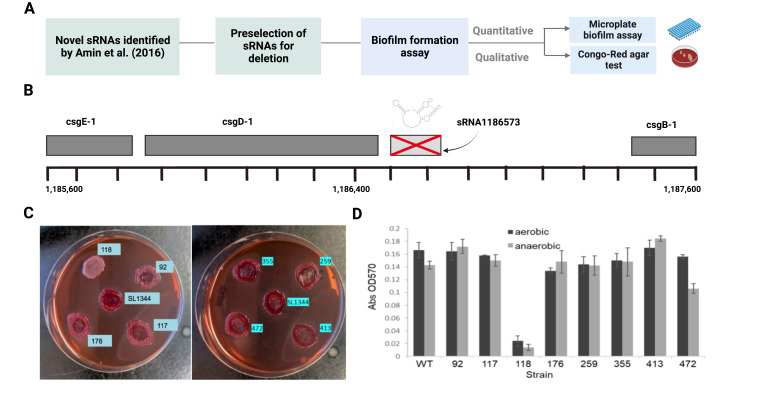
(A) Generalized workflow depicting the experimental procedure. (B) Generation of deletion mutants by lambda red recombination. sRNA1186573 was used to represent the mutation generation event. The best thermodynamically stable secondary structure of sRNA1186573 (predicted by Mfold (Zuker, 2003)) is placed above the respective sRNA sequence (light grey). Names and locations of neighboring genes befalling within the 2kb region as defined in the Ensembl (Zerbino et al., 2018) build are included in dark grey. (C) Morphotypes of deletion mutants on Congo red agar, 72-h at 37°C. Here all sRNA mutants show 'rdar' morphotype (red, dry, and rough; expresses curli and cellulose) except the sRNA mutant 1186573, which shows a ‘saw’ morphotype (smooth and white; neither curli nor cellulose). SL1344 is the wild type of Salmonella enterica serovar Typhimurium strain. (D) Microplate biofilm assay, 24-h at room temperature. Deletion of sRNA 1186573 had a lower optical density at 570 nm compared to its counterparts in both aerobic and anaerobic conditions. sRNA names are abbreviated to fit within the figure (e.g., 118 is an abbreviation of 1186573).

## Description

*Salmonella enterica* is rod-shaped, motile, non-spore-forming gram-negative bacilli responsible for causing gastrointestinal disease and thousands of deaths across the world every year. Salmonella, like many other bacteria, forms biofilm when subjected to stress (Harrell et al., 2020) . Biofilms are surface-attached communities of bacteria surrounded by self-produced extracellular polymeric substances (Costerton et al., 1999; Flemming et al., 2016) which are formed as a result of exposure to unfavorable external stimuli from the environment (O’Toole et al., 2000). Biofilms act as a “fortress” by protecting the bacteria from harsh external stress, such as antibiotics. This makes biofilms an area of concern in the medical field because of the resistance it provides to bacteria (Harrell et al., 2020) .

Small RNA (sRNA) are non-coding RNA molecules that are less than 200 nucleotides in length and have been found to control gene expression in many regulatory circuits in bacteria, such as *Acinetobacter baumanii* , which is a classic biofilm former (Sarshar et al., 2022) . Having previously identified sRNAs putatively targeting regulators of biofilm formation in *E.coli* and *S. enterica* : *bssR* and *csgD* (Amin et al., 2016) , in this study we attempted to identify sRNAs directly contributing to biofilm formation in Salmonella (Jørgensen et al., 2020).

To examine the relevance of specific sRNAs on biofilm formation in *Salmonella enterica* serovar Typhimurium, we selected eight unique sRNAs for deletion primarily based on their genomic location and/or putative targets then performed qualitative and quantitative assays of biofilm formation on each mutant and controls ( **Fig. 1A, 1B** ). After 24-h of incubation at room temperature in a nutrient deficit condition (as nutrient starvation is an inducer of biofilm formation (Mika & Hengge, 2014) ), we observed normal “rdar” morphology (normal curli protein and cellulose expression) for all the deletion mutants except for the sRNA1186573 deletion, for which curli protein and cellulose expression were significantly impaired (as determined by Congo Red assay and established biofilm morphotype) (Römling et al., 1998; Zogaj et al., 2001) ( **Fig. 1C** ). In agreement with Congo Red assay results, standard microplate biofilm UV absorbance assays confirmed that the ability of the sRNA1186573 deletion mutant to form biofilms during nutrient deprivation was almost entirely lost, whereas the ability of the other sRNA deletion mutants to form biofilms did not significantly differ from wild type ( **Fig. 1D** ).

In conclusion, our results confirm that sRNA1186573 is required for efficient biofilm formation in *S. enterica* , further highlighting the significance of sRNAs during Salmonella stress response. Finally of note, although RT-qPCR analyses confirm that sRNA1186573 deletion does not disrupt *csgD* gene expression, it is tempting to speculate that this sRNA may be involved with the post-transcriptional regulation of *csgD* , as csgD protein directly contributes to curli expression in *Salmonella enterica* (Amin et al., 2016; Bokranz et al., 2005) .

## Methods

**Pre-selection of sRNAs and mutant generation**

A total of 8 sRNAs (i.e., 924744, 1170414, 1186573, 176086, 2594511, 3551252, 4130247, and 4720054) in *Salmonella enterica* serovar Typhimurium (Borchert et al., 2009; Borchert, Holton, & Larson, 2011; Devadoss et al., 2022) were pre-selected from our initial published list of novel Carbon starvation responsive sRNAs (Amin et al., 2016; Houserova et al., 2021) based on (1) informatic establishment of dynamic expression (Borchert et al., 2009; Borchert, Holton, & Larson, 2011; Devadoss et al., 2022) , (2) genomic context in proximity to (but not overlapping) biofilm relevant genes (Borchert et al., 2006; Borchert, Holton, Williams, et al., 2011; Cardin & Borchert, 2017; Chevalier & Borchert, 2017; Ehrat et al., 2012; King & Borchert, 2017; Roberts et al., 2013, 2014) , (3) and/or biofilm relevant predicted gene targets (Chen et al., 2018; Coley, Stahly, et al., 2022; Coley, DeMeis, et al., 2022; Filshtein et al., 2012; Huang et al., 2016; Roberts et al., 2018; Roberts & Borchert, 2017) . SL1344 deletion mutants of the selected sRNAs were generated using the Lambda-Red recombination method (Barnhill et al., 2019; Murphy, 1998; Spector & Kenyon, 2012) . Briefly, lambda-red recombination genes from the pKD46 plasmid were induced with arabinose in wild-type SL1344 cells. The pKD3 plasmid was used to amplify the chloramphenicol resistance cassette flanked by sequences corresponding to the sRNAs selected for the knockout. The resulting mutant strains were confirmed by colony PCR. Δ92, an sRNA deletion mutant we previously generated using the same methodology (Amin et al., 2016) was utilized as an sRNA deletion control. Conditions and cycling parameters were performed exactly as previously reported by our laboratory (Barnhill et al., 2019) .

The primers used for generating mutants are mentioned below:

sRNA924744 F: CACATTCACCGCTTACACAGGTCTGAACAAGGGGAGGCGAGTGTAGGCTGGAGCTG

sRNA924744 R: AAGGCTCCAGTATATTTTTAAAGGATTTTTGGCATAATGAACATATGAATATCCTCCT

sRNA1170414 F: GTAGTAATAGCGGTAGTTCCCCGGCAGTGATGGTCACTCAGTGTAGGCTGGAGCTGC

sRNA1170414 R: AATGATGAGAGCTTTTAAGATGACAAGACCACCACCGGCGACATATGAATATCCTCC

sRNA1186573 F: GTAATGGCTAGATTGAAAACAGTTAGTGTAGGCTGGAGCTGCTTC

sRNA1186573 R: CCCCATAAAATAAAGGCACCAGAAGTACTGACAGATGTTGCATATGAATATCCTCCTT

sRNA176086 F: TGAATTTGACACTGCGCACAGGGCGA

sRNA176086 R: ACGACCTGCTTCTGAGGCTTTCTCTTT

sRNA2594511 F: AAATAAGATCCCGGCCAGCCTGATAC

sRNA2594511 R: CGTGAACTGGGGAACTGGAAAGATTT

sRNA3551252 F: TTTTAATATCATTAAAATCAAAAGTATAGACATTCATAGCGTGTAGGCTGGAGCTGC

sRNA3551252 R: TGACTATACTTATTTGAGATACAAAAACAGCGCAAGAGTGCATATGAATATCCTCCT

sRNA4130247 F: ATCTTGTGCTATTGGCAAAACCTATGGTAACTCTTTAGGTGTGTAGGCTGGAGCTGC

sRNA4130247 R: TCGTCCAAGTGCAGCCCCGCACGGTGGGATAATAATCACCACATATGAATATCCTCCT

sRNA4720054 F: CACAAAACTTATGGATTTATGCGTATAATCCGCGGCGCAAGTGTAGGCTGGAGCTGC

sRNA4720054 R: CGTTATTGTGTCACTGTCTTACACACCGGTAAGACAGCAGACATATGAATATCCTCC

The primers used for confirming the mutants are here below:

sRNA924744 F: GAATCCCCAGCAAACCAAG; sRNA924744 R: GCAGGCATAGTGATGATTTCC

sRNA1170414 F: CTATGGAGATCGCGAATGGT; sRNA1170414 R: GAATGTCCGTACAGGGTGTTG

sRNA1186573 F: AGGCACCAGAAGTACTGACAGA; sRNA1186573 R: ACGGCTATTTCAACCCACAG

sRNA176086 F: GACATATCATATTTAAAACGCAACA; sRNA176086 R: CGCGATGTTCTGCCATAAT

sRNA2594511 F: TCTTCGTTGAGTCGCCTTT; sRNA2594511 R: CGTAAATAAATGCCTGGAAGG

sRNA3551252 F: ACCATCCCGACAGACAA; sRNA3551252 R: TTGGAAGTGAAACCTCTGCAT

sRNA4130247 F: AGCCAAGATGCAAGAATAGACA; sRNA4130247 R: CCACGCTAATCACGACCA

sRNA4720054 F: TTACTTACCGGAGGCGACAT; sRNA4720054 R: GAAAATTCTCCATCGCGG

The primers used to confirm that sRNA1186573 deletion does not disrupt *csgD* gene expression are as follows:

CsgD_F_qPCR: GGTCAGCGGATTACAGGGTA; CsgD_R_qPCR: TCGCGATGAGTGAGTAATGC

**Biofilm formation assay**

Both qualitative (Congo-Red agar test (Milanov et al., 2015) ) and quantitative (Microplate biofilm assay) assays of biofilm formation assays were performed. Congo Red agar (CRA) plates were prepared from yeast extract (5g/L), Bacto Tryptone (10 g/L), technical agar (15 g/L), Congo Red (40 mg/L), and Coomassie brilliant blue (20 mg/L). Overnight cultures were locally spread on the plates which were incubated at room temperature (RT) for 72 hours. Results were defined based on the following morphotype traits previously established in *Salmonella Typhimurium* : (i) rdar (red, dry, and rough; curli and cellulose expression), (ii) pdar (pink, dry, and rough; cellulose expression), (iii) bar (brown, dry, and rough; curli expression), and (iv) saw (smooth and white; neither curli nor cellulose) (Cimdins & Simm, 2017) , saw / mucous (capsule production) and less-obvious phenotype explained by Bokranz *et al* . (Bokranz et al., 2005) : ras (violet and smooth; curli only), bas (brown and smooth; curli only) or pas pink and smooth; cellulose only). In addition, each mutant was also cultured in diluted Tryptic-Soy broth (TSB) (1/20) overnight on a shaker at 37°C for the microplate biofilm test. 20 µl of the culture and 130 µL of TSB were placed in individual 96-well plate wells. Following inoculation, the plate was either sealed (for anaerobic conditions) or pierced in the middle of each well to allow for oxygen exchange (for aerobic conditions) after which the plate was placed at RT for 24 hours without shaking. After discarding the inoculum, each well was washed three times in 200 µl of RO water. 170 µl of 75% ethanol was then added, and the plate was incubated for 10 minutes at RT. The ethanol was removed, and the plate was dried at 37°C for 20 minutes. 170 µl of 75% ethanol was then added to each well, and the plate was incubated for 10 minutes at RT. Next, the ethanol was removed, and 170µl of 0.1% Crystal Violet was added to each well and left to incubate at RT for 10 minutes. After this, the ethanol was removed, and the wells were gently rinsed with deionized water. Finally, 170 µl of acetic acid was added to each well, and an ELISA plate reader was used to read absorbance at OD570.
